# Auditory perceptual discomfort and low-hearing tolerance in the first episode psychosis

**DOI:** 10.1186/s41155-022-00224-0

**Published:** 2022-07-11

**Authors:** Maria Lúcia de Bustamante Simas, Naianna Ribeiro Mocelin dos Santos, Aline Mendes Lacerda

**Affiliations:** Laboratório de Percepção Visual, Programa da Pós Graduação em Psicologia, Departamento de Psicologia, Centro de Filosofia e Ciências Humanas, Universidade Federal de Pernambuco, Av da Arquitetura s/n CFCH 9º Andar, Recife, PE CEP.: 50740-550 Brasil

**Keywords:** FEP, Hearing tolerance, Hearing discomfort, Auditory sensitivity, Pure-tone frequency sweep

## Abstract

*Awareness* of perceptual and sensory changes that might occur in visual, auditory, proprioception, and other senses, in the early stages towards the First Episode Psychosis (FEP), and their subsequent sensorial evolution as the disturb progresses deeper into an acute episode, might be a key element for interrupting the process. In the present study, we investigated hearing discomfort/tolerance to 16 given sound streams. Sixteen people diagnosed with FEP, participated in the experiment. Sixteen frequency sweeps varying in modulation envelopes (sawtooth, sine), order (ascending, descending), duration (4s, 8s), and range (50–8000 Hz, 2–8 kHz) were presented randomly, but always in the same sequence, to FEP and healthy controls (HC). The level of discomfort was estimated by the participant by making a mark across a continuous line whose extremes read “nothing bad” (left) and “too bad” (right). Results showed that ascending sine pure frequency sweeps (*p* < 0.01) and descending sine pure frequencies sweeps (*p* < 0.01) caused the maximum discomfort in FEP. Other variables also showed differences between FEP and HC, and FEP were always more intolerant to such pure frequency sweeps than HC. We conclude that this might be useful for very early assessment of people at risk, people with FEP, and people with schizophrenia.

## Introduction

The first episode psychosis patients (FEP) are generally unaware of perceptual alterations in the prodromal and acute states of the disease they experience. Awareness of the changes that might occur in visual, auditory, proprioception, and other senses and that evolve as the disturb progresses deeper into the prodromal, or further in the acute phase, can be a powerful tool for interruption of this process, and eventually, for prevention of future acute episodes in FEP and schizophrenia.

In previous studies on vision, we have observed a tendency of people with schizophrenia (and depression) to easily perceive exceptionally large pictorial images occurring in everyday natural scenes as well as a tendency to selectively perceive objects of larger or wider magnitudes as more salient in the surrounding environment (de Bustamante Simas et al., 2011; Lacerda et al., 2020). This finding has been repeatedly reproduced in our studies with volunteers diagnosed with schizophrenia interns (or not) in psychiatric hospitals, or clinics, attending day-clinics, or seeking ambulatory services, mostly in remission and always medicated (de Bustamante Simas et al., 2021).

In the present study, we focus our attention on first-person accounts (one of the authors included) of empirically observed hearing alterations in schizophrenia that differ from those commonly found in the literature, namely, studies on hearing voices, measurements of mismatch negativity (MMN), and auditory steady-state response (ASSR) to 1–2kH pure tones at 40-Hz stimulation rate. Thus, these empirical observations include the noticeable increase in sensitivity to auditory stimuli of very high pitch like motor vehicle strident sound alarms, or the sound made by cicadas and crickets close to sunset, as well as to stimuli of very low pitch and constant vibration, like the noise made by air conditioner compressors or fans. Such stimuli appear to be perceived as very salient in the surrounding environment by people with schizophrenia, mainly in prodromal or acute states. To better contextualize our study, in this introduction, we make a brief review of the available and indirectly related literature and proceed to the present our view and hypothesis.

The peculiar event-related potential responses (ERP) to the auditory oddball paradigm first found by Butler (1968) in habituation experiments and subsequently reported by Squires et al. (1975) was latter observed to be abnormal in people with chronic schizophrenia (Duncan et al., 1987; Duncan-Johnson et al., 1984). This finding yielded a wealth of studies that resulted in the proposal of having the measurement of MMN (that is not a component from ERP in itself, but a difference resulted from the subtraction between ERPs to a standard stimulus and that to an odd/rare stimulus) as a biomarker for schizophrenia (e.g., Light & Näätänen, 2013; Nagai et al., 2013). Yet, measurements of MMN for FEP did not consistently produce the expected evoked potential MMN deficiency (e.g., Salisbury et al., 2017). Nevertheless, more recently, Curtis et al. (2021) measured the volume of the auditory and the inferior frontal cortices of FEP spectrum and argued that pitch MMN and duration MMN can indeed be biomarkers of the underlying pathological deficits in schizophrenia.

Although well established as a potential biomarker, the exact relationship of the MMN with the actual sensory perception is not clear. In 2016, Haigh et al. pointed out to three hypotheses: a memory-based model from Näätänen (1990), a predictive model with basis on the subject’s expectation from Winkler (2007), and that from May and Tiitinen (2010). The latter being that there are increased sensory responses to rare deviants in relation to repeated sensory-adapted stimuli. In other words, these authors assume that there is an adaptation effect to the standard stimulus set and a specific relatively enhanced response to the deviant stimulus, whose characteristics strongly influences the N_1_ component from its respective auditory ERP and, therefore, affecting the magnitude of the MMN response (resulted from the subtraction between the two ERPs). Indeed, Adler et al. measured auditory ERPs to repeated 1kHz pure tone stimulus, varying in duration and stimulation frequency, with schizophrenic patients and found that there is an increase in N_1_ amplitude, and latency, at stimulation rates of intertrial intervals between 1 and 2s (Adler et al., 1990). Previously, Roth et al. had found that N1 amplitudes get smaller in schizophrenics as stimulation intertrial intervals get longer (Roth et al., 1980). Besides, in 1994, Salisbury et al. found that, in an odd ball paradigm, auditory stimulus characteristics and discriminability affected P3 latency, but not amplitude, in people with schizophrenia (Salisbury et al., 1994), and this fact also impacts the MMN response, as does the deviant stimulus intensity, duration, and frequency (Todd et al., 2008).

We side with the hypothesis from May and Tiitinen (2011) mentioned above and illustrate with an example where a FEP patient recently medicated asked “what noise was that?” when a voltage shift, suddenly, changed the velocity and noise emitted by the motor of an air conditioner fan during attendance by a psychiatrist. This can be interpreted as an oddball paradigm or MMN event. The patient noticed and adapted to the sound of the fan when an instantaneous noise discontinuity/change occurred in midst of sensory adapted stimuli. A shift in noise often ignored by people in general. The exact behavior of the auditory ERP, as well as the different aspects of the MMN (i.e., simple, pitch, or duration MMN) for that given patient, under those particular circumstances, remain to be assessed.

In a similar vein to MMN, ASSR generated another strain of studies, including that of possible interrelationships between them both (Koshiyama et al., 2018). A meta-analysis, from 1999 to March 2016, also endorses its measurements as a schizophrenia biomarker (Thuné et al., 2016). As for the MMN, different characteristics of the ASSR are suggested to be related to different aspects of the disease and, therefore, useful for clinical assessment (Griskova-Bulanova et al., 2018; Manting et al., 2020). Still more recently, Coffman, working with the group of Salisbury (Coffman et al., 2022), found that FEP patients with auditory hallucinations (AH) were unable to modulate (increase) their ASSR response with the manipulation of attention within an attend-ignore paradigm. These authors conclude that there seems to be a deficit in a cognitive control of auditory neurophysiology in these FEP patients with AH, probably because an hyperexcitability of the auditory cortex, and suggest the likely possibility of an oversensitivity to auditory sensory response.

So, currently, we work with the hypothesis of FEP having increased sensitivity to odd and salient auditory stimuli within the environment. This would be mostly likely due to increased sensory response sensitivity to certain ranges of sound stimulus pure frequencies. Also from empirical observation, we assume this might be true for odd and salient transient brightness stimuli (such as a sudden and transient solar ray reflection) in the case of visual perception (though this is not under scrutiny here).

Thus, for the purpose of this study, we investigated the broader hypothesis of increased auditory sensitivity in people with schizophrenia in the literature while focusing on psychophysics. We only found qualitative studies on the hearing perception of schizophrenic patients (Freedman & Chapman, 1973; Landon et al., 2016). But we found almost no works psychophysically evaluating sensitivity to pure tone frequencies in schizophrenia. Yet, some of the more recent studies (mostly on varying loudness perception, MMN aspects, some on Tinnitus) do measure audiometric functions as a standard practice, but do not investigate the possibility of increased sensitivity to specific pure frequency ranges in patients with schizophrenia. They do check for “normal hearing” or “non-abnormal hearing,” “no hearing impairments” (e.g., Atkinson et al., 2012; Curtis et al., 2021; Hsieh et al., 2019; Iliadou et al., 2013; Prager & Jeste, 1993; Salisbury et al., 2017). One study, for instance, defines “normal hearing” as “within 30 dB *n*HL (and) no more than 15 dB difference between ears at 500, 1000, and 1500Hz” (Salisbury et al., 2017).

But when we examine the literature, tables presented in some of the studies do show some evidence of increased sensitivity in schizophrenic patients for given pure-tone frequencies. Nam (2005), for instance, reports high-hearing sensitivity for schizophrenic patients n. 3, 4, 6, and 12 in Table 1 (p. 353), for ears right and left, when describing “5f-PTA (dB)” thresholds, that is, “5 frequency-pure-tone average hearing threshold = (0.5+1+2+4+8kHz)/5”. We observe that for those patients, these average thresholds are below 5 dB, in strong contrast with other subjects. In that same direction, Lliadou et al. (2013, Table 1, p.203) present pure-tone audiograms (PTA) measured for schizophrenic first episode patients (right and left ears) whose thresholds are about 1.5-fold below the control group at the 2000 Hz frequency. While the former work addresses the issue of hallucinations and Tinnitus, the latter raises questions on the existence of “central auditory processing disorder” employing a psychoacoustic methodology and a test battery based on the American Academy of Audiology (2010, as cited by Iliadou et al., 2013). However, such tests involve mostly speech and speech-like sounds, not pure-tone frequencies (Iliadou et al., 2013, Table 2, p. 204).

**Table 1:** Sample characteristics

Parameters	HC	FEP
**Gender**	14 men	16 men
**Mean age (SD)**	27.86 (10.02) years	25.57 (8.38) years
***p*value (Mann-Whitney*U*test)**	*p* = 0.490	
**Mean education (SD)**	11.71 (2.52)	10.39 (3.12)
***p*value (Mann-Whitney*U*test)**	*p* = 0.232	

*Note. SD* standard deviation. Years of education were counted as follows: fundamental incomplete = 4,5, fundamental = 9, high school incomplete = 10,5, high school = 12, university degree incomplete = 14, and university degree = 17. There were no significant differences between the samples

**Table 2:** Beta regression coefficient estimates (for *N*=32) and respective *p* values for all factor levels, as well as for combined ascending (or descending) sine (or sawtooth) envelope modulations. Please observe that negative values are contextual to the statistical analysis procedure and do not have specific attached theoretical meaning in this case

Factor levels	Estimate	Standard error	***p*** value
**ASC STH**	−0.275	0.149	0.0656
**ASC SINE**	−0.347	0.132	0.0085**
**DESC STH**	0.004	0.159	0.9816
**DESC SINE**	−0.410	0.143	0.0042**
**STH**	−0.277	0.135	0.0403*
**SINE**	−0.325	0.138	0.0183*
**ASC**	−0.313	0.133	0.0185*
**DESC**	−0.301	0.142	0.0342*
**0.5**−**8 kHz**	−0.214	0.152	0.1596
**2**−**8 kHz**	−0.278	0.136	0.0411*
**4 s**	−0.266	0.156	0.0889
**8 s**	−0.338	0.144	0.0187*

*Note.* Difference between groups *p < 0.05; **p < 0.01

Both, the works of Nam (described above) and Dölberg et al. (2008) addressed the issue of Tinnitus in patients with schizophrenia. Nam measured pure-tone hearing levels of 15 patients with hallucination and/or Tinnitus, and only seven of those had tinnitus. He found that only hallucination was related to abnormal brain stem responses and concludes that hallucination and tinnitus should be differentiated among schizophrenic patients. Three of those patients hallucinating and one having only tinnitus showed very high hearing pure-tone sensitivity. But, from Nam’s paper, it is not possible to establish the exact range of maximum hearing sensitivity for those patients, nor the range of tinnitus. Dölberg et al. (2008) examined 31 patients suffering from Tinnitus and found that it occurred mainly at frequencies between 3 and 8 kHz, being the most frequently observed that in the range between 5 and 6 kHz. From their Fig. 1 (p. 720), we assume they did not test frequencies above 8 kHz. In 2016, Sanchez et al. found that Tinnitus is associated to reduced tolerance to sound level in healthy adolescents from 11 to 17 years old. Their audiogram (as seen on their Fig. 2, p.4) present high sensitivity hearing levels at 14–16 kHz besides the range of 2–4kHz. From 170 participants, 54.7% reported having experienced Tinnitus. But, even in those cases, we should observe that tinnitus is not clearly, and not easily, differentiated from exceedingly high hearing sensitivity.

**Fig. 1:**
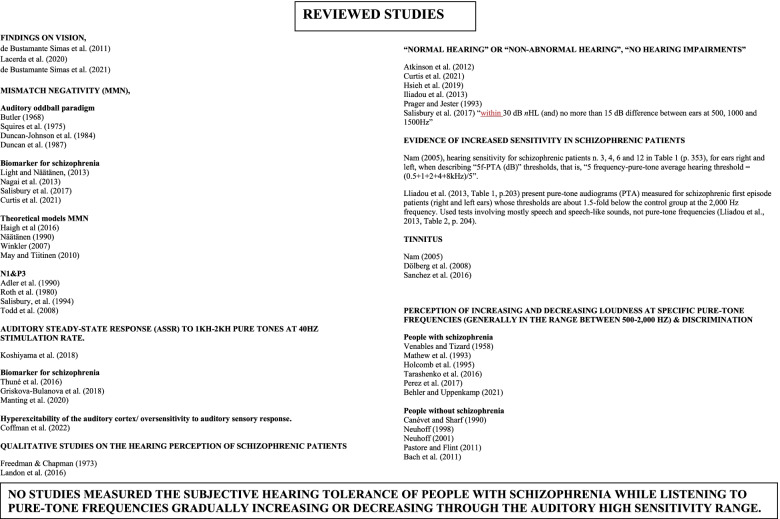
A brief summary of the reviewed literature

**Fig. 2:**
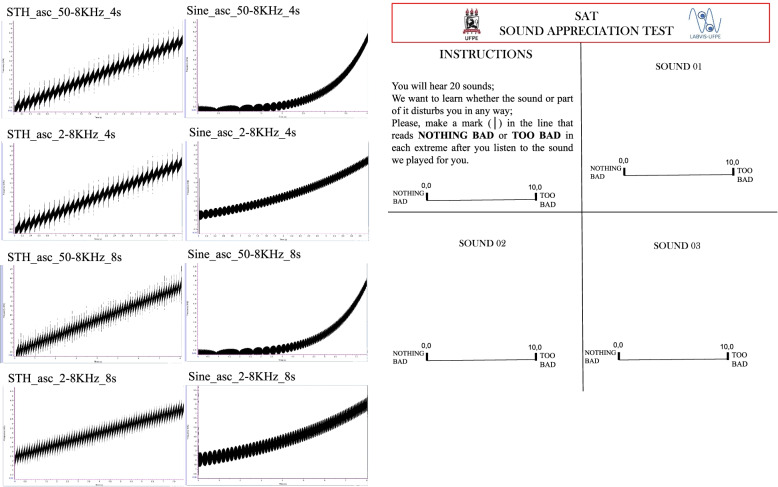
Ascending soundwave spectra [extracted through the software **Raven Pro 1.6** by Michael Pitzrick from Cornell University] (left) and an illustrated example of pages from the response pad (right)

On the other hand, a few studies have systematically investigated perception of increasing and decreasing loudness at specific pure-tone frequencies (generally in the range between 500 and 2000 Hz) as well as discrimination between pure-tone frequencies. Among these are the works of Venables and Tizard (1958), Mathew et al. (1993), Holcomb et al. (1995), Canévet and Scharf (1990), Neuhoff (1998), Neuhoff (2001), Pastore and Flint (2011), Bach et al. (2011), Tarashenko et al. (2016), Perez et al. (2017, b), and Behler and Uppenkamp (2021). But only the first three and the last three works involved schizophrenic patients.

Venables and Tizard, contrary to their initial hypothesis of a paradoxical effect based on earlier visual studies (1956; 1958), found decreasing reaction times with increasing auditory stimulus intensities in schizophrenia patients, as normally would be expected.

Mathew et al. (1993) measured auditory acuity for pure-tone frequencies (250–8000Hz) in hallucinator and non-hallucinator chronically ill schizophrenic patients (29–64 years old, mean 50) as compared to healthy controls. And found that lateral asymmetry for hearing between ears correlated with hallucinators in comparison to non-hallucinators and controls. Interestingly, acuity for schizophrenics was found to be lower for frequencies above 0.5–1 kHz. These authors did not consider the possibility of either the presence of Tinnitus, or hypersensitivity hearing, in any range of frequencies that could interfere in detection due to masking effects, nor did consider the possibility of attention focus difficulties since tones could be presented to either ear and depended on the volunteer raising the right or left hand.

Holcomb et al. (1995) measured discrimination between 800 and 1500 Hz compared to the discrimination between 1300 and 1500 Hz at 69, 72, 76, and 83 dB stimuli, always lasting 100 ms in duration. Participants had 2500 ms to respond or would miss the trial. Controls (*n*=9) were more accurate than schizophrenic patients (*n*=11). As loudness was increased, missed trials were reduced for schizophrenics. For controls, this was an invert U shape. Audiometry was not reported.

Bach et al. (2011) measured the perception of loudness change by schizophrenic patients for sounds of rising intensities by changing, directly with the computer mouse, the value of a “visual analog scale” defined by a horizontal line whose extremes read “no change”-“high change.” Previous findings had found bias towards higher sensitivity to ascending sound intensities (when compared to descending), even at the same ranges, in healthy volunteers (Neuhoff, 1998). Bach and coworkers do not specify the pure-tone frequency(ies) adopted, only duration of 2000 ms and intensities. But they conclude that schizophrenics have “impaired extraction of meaning for dynamic sound intensity” perception because they do not estimate changes in intensity (looming and receding) as linearly, neither as intensely, as controls (Bach et al., 2011, Fig. 1). It seems that volunteer schizophrenic patients, in this study, did not interpret increasing amplitude as movement or displacement in space. These authors do not consider the possibility of increased loudness sensitivity to high or/and low frequency ranges, in schizophrenia, that could incur in diminished sensitivity to spatial localization depending on the selected frequencies and other specific characteristics of the stimulation. Impaired sound localization should also be considered (Perrim et al., 2010).

Tarashenko et al. (2016) and Perez et al. (2017, b) evaluated cognitive abilities after training discrimination between “two-tone” sound “sweeps” of various frequencies (and varying interstimulus intervals) organized in terms of increasing difficulty. They found, among other things, that the number of training levels completed did not correlated well with auditory attention isolated. Nevertheless, the measurement of MMN was sensitive to such training (Perez et al., 2017, b).

Pastore and Flint (2011) measured magnitude judgements of loudness changes with healthy volunteers. They argue that subjective loudness is a complex attribute of sound because it “is not equivalent to power, relative power, or dB change.” Their purpose was to investigate the finding that ascending loudness is perceived as higher than descending loudness in the equivalent frequency range as previously reported by Canévet (e.g., Canévet & Scharf, 1990) as well as Neuhoff (1998, 1999, 2001). They conclude that perceived looming is dependent on onset and offset reference tones and is not a simple relationship.

Behler and Uppenkamp (2021) report evidence with health volunteers for involvement of the orbitofrontal cortex and medial temporal areas in the judgement of dynamic stimuli ascending and descending loudness. This finding favors the hypothesis of cognitive involvement in the processing of varying loudness and does support, in part, the conclusion previously assumed by Bach et al. (2011) regarding the perception by schizophrenics.

Despite the considerable number of studies on many aspects of hearing carried with schizophrenic patients, no studies measured the subjective quality of such perception when pure-tone frequencies are gradually increased or decreased. Our empirical observations suggest reduced tolerance associated with that sort of stimuli in people with schizophrenia, mostly enhanced in prodromal and acute states.

Bearing in mind those reviewed works (refer to Fig. 1), our current experiment used pure-tone frequency sweeps ranging from 50 to 8000 Hz lasting 4 or 8 s (constant amplitude) and measured hearing tolerance as the level of subjective discomfort ranging from “not bad” to “very bad” along a continuous horizontal line showing zero (0) on the left side and ten (10) on the right side. Thus, ascending, or descending, sweeps of pure-tone frequencies (modulated by saw-tooth or sinewave envelops) were presented to volunteers, our hypothesis being that the observed sound discomfort level (SDL) would be higher for the FEP group when compared to a matched control group.

## Method

### Participants

A convenient pseudo-random sample of 30 volunteers, 16 volunteers (18–50 years old, 16 men) attending the ambulatorial service at PEP/HC/EBSERH/UFPE composed the first episode psychosis group (FEP), and 14 diagnostic-free healthy participants tentatively matched to the experimental group for gender, age, and educational level composed the healthy control group (HC). For statistical treatment and equal *N* in the two samples (i.e., *N*=16), we used the mean from the 14 HC to add two more cases to this latter group.

### Inclusion criteria

FEP: (1) diagnosed according to ICD 10 as F23, F20, F30.2, F32.3, or F22, all within 1 year of symptoms; (2) attending medical service for *first episode psychosis patients* at PEP-HC/EBSERH/UFPE, all of them making use of atypical antipsychotic medication; (3) normal or corrected to normal visual acuity; and (4) older than 18 years old.

HC: (1) diagnostic free of neuropsychiatric diseases; (2) normal or corrected to normal visual acuity; and (3) older than 18 years old.

Unfortunately, though initially planned, we could not perform an audiometric screening of volunteers prior to the experiment because of the noisy surround of the *on-site* ambulatory experimental set up (used for the FEP group). But no participant of either group showed any apparent, or complained of, existing hearing deficits. We believe all volunteers were well within the definition of “normal hearing” used by (Coffman et al., 2022; Salisbury et al., 2017), i.e., “within 30 dB *n*HL (and) no more than 15 dB difference between ears at 500, 1000, and 1500Hz.”

### No-inclusion criteria

FEP: (1) diagnosis of secondary dementia associated with the first episode psychosis and (2) neuromuscular-osteopathic or other neurological diseases.

HC: (1) neuromuscular-osteopathic or other neurological diseases and (2) regular use of neuropsychiatric medication.

#### Instruments

For initial assessment of the mental and cognitive status, we used the Standardized and revised Addenbrooke’s Cognitive Examination, ACE-R (Amaral-Carvalho & Caramelli, 2007). The ACE-R was found by Brazilian researchers, de Souza and colleagues (e.g., de Sousa et al., 2021), to be more sensitive (and to show a better performance) as well as accuracy, in discriminating patients according to both variables, level of education and cognitive ongoing state, than did the Mini Mental state Examination (MMSE), and the Montreal Cognitive Assessment (MOCA), therefore being the choice of this hospital service for brief cognitive assessment at admission.

The experiment also involved the following instruments:

- Cel phone for sound stimuli presentation at ~ 65 dB.
- Tone Generator/Wavepad/Mixpad NCH software for sound editing and mixing.
- Pure-tone frequency sweep stimuli for the Sound Appreciation Test, SAT, that were produced with NCH software: 8 linear frequency sweeps with sawtooth (STH) envelopes (34 steps) and 8 logarithmic frequency sweeps with sine (SINE) envelopes (quasi-continuous).The sixteen frequency sweeps were from 50 to 8000Hz (*n*=8) or from 2000 to 8000Hz (*n*=8), with durations of 4s or 8s each (*n*=8, respectively), being 8 ascending (ASC) and the same 8 descending (DESC) (Fig. 2, shows spectra of ASC only stimuli)
- Response pad with instructions, sounds numbered from 1 to 16, and lines sided by NOTHING BAD on the left and TOO BAD on the right side of a 10-cm horizontal line, according to Fig. 2.

#### Procedure

Upon agreement from the FEP medical service at Hospital das Clínicas/EBSERH/UFPE, Recife, PE, Brazil, and submission to the Ethics Committee (Plataforma Brasil-CAEE-n. 23665419.5.0000.8807), we began the individual experimental sessions always in the same sequence with FEP and HC. All volunteers read and signed consent forms. At the time the experiments were run, 10 FEP patients were with the hospital service for about 1 year, three other FEP patients for 6 months, and yet another one for 1 month only.

After a thorough explanation of the procedure and assurance of the full understanding of the steps to be taken, the participant signed the consent form. This was followed by a semi-structured interview to record personal and familial medical history, and by the Addenbrooke Cognitive Examination, ACE-R. Only then, the Sound Appreciation Test (SAT) was initiated by the instruction “You will hear 16 sounds. We want to learn whether the sound or part of it disturbs you in any way. Please, make a mark (│) in the line that reads NOTHING BAD or TOO BAD in each extreme after you listen to each sound played for you”. The volunteer should make a mark along the horizontal line presented in a pad with a sequence of sheets numbered from 1 to 16 (refer to Fig. 2), one sheet per sound number. The complete procedure lasted less than 50 min on the average.

#### Raw data handling

Continuous values within the interval of 0–10 cm attributed by the volunteers as estimates of SDL were organized by stimulus modulation envelope (Sawtooth, Sine), order (ascending, descending), duration (4s, 8s), and range (0.050–8kHz, 2–8kHz), per group, per volunteer. Since the control group (HC) had only 14 participants, we completed the sample adopting the values of the mean for the 14 participants from HC to equal *N* (*N*=16) in both samples, FEP and HC.

Statistical analyses were carried out with the software Statistica 14. The Mann-Whitney *U* test was used for assessing differences in age, education, and the Addenbroke Cognitive Examination between the independent groups.

### Principal component analysis (PCA) and beta regression

For dimensionality reduction, principal component analysis (PCA) of the observed values were carried out for each of the eight factor levels per group. Weights were extracted based only on the first principal component. Raw sample values were then weighted, normalized based on the maximum possible attributable value (i.e., 10), and converted to a distribution varying between 0 and 1.

Following this procedure, we used beta regressions with both samples taken together to evaluate significance of the observed differences between groups for each of the given stimulus factor levels because “beta distributions are very versatile, and a variety of uncertainties can be usefully modelled by them. This flexibility encourages its empirical use in a wide range of applications” (Johnson et al., 1995, p. 235). The main reason for this choice was the small *N* size of the samples.

### Correlations between collected/observed data

We also used the Spearman Rank to test correlations among the volunteers’ responses to the 16 sound stimuli as well as between those and age, and education and the Addenbroke Cognitive Examination scores.

## Results

### Beta regression analysis

**Fig. 3:**
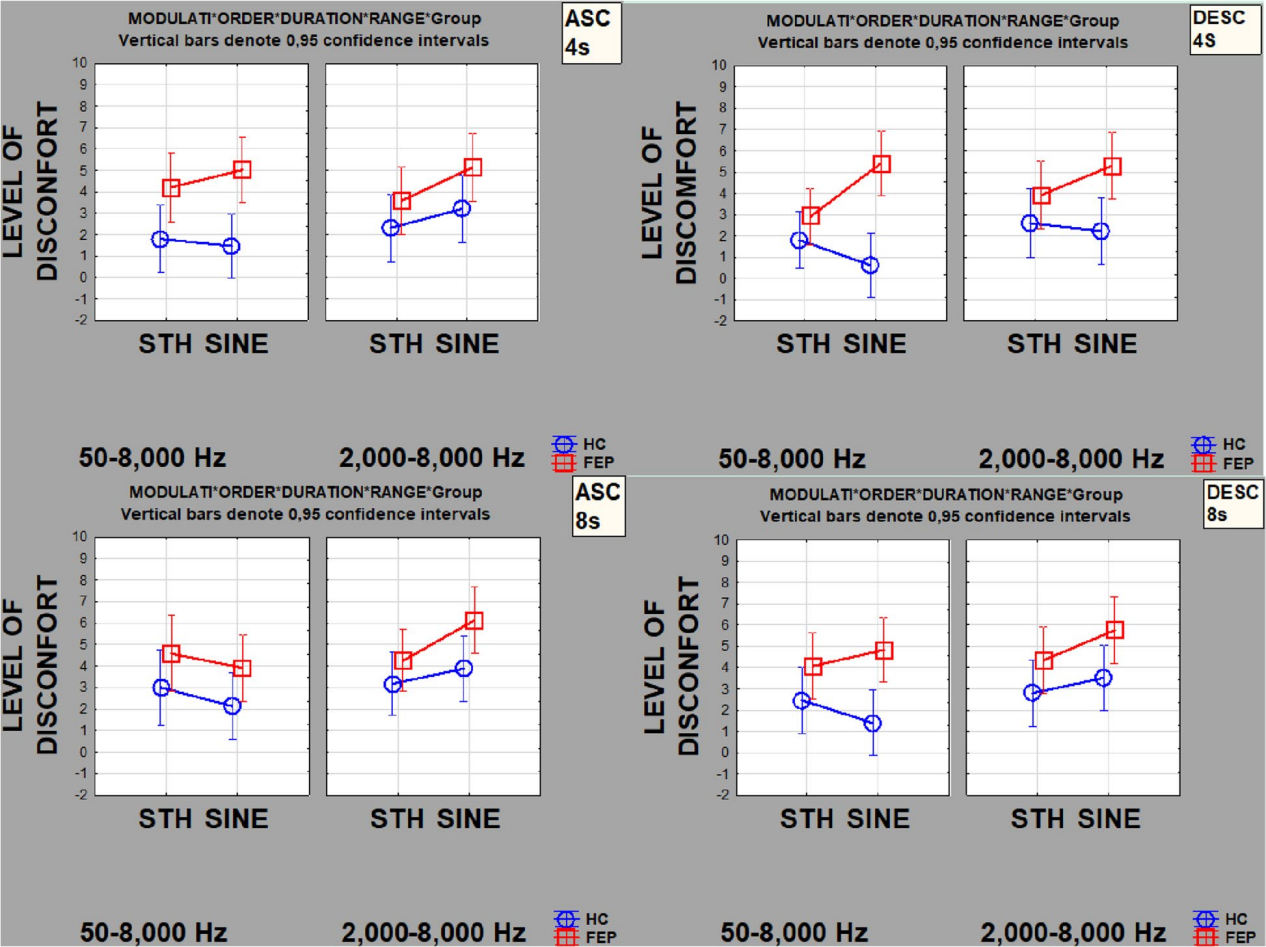
Observed SDL for FEP (red-square) and HC (blue-circle). Note that in three conditions (SINE, 4s, 50–8000 Hz, ASC, & DESC; and SINE, 8s, 50–8000 Hz, ASC), the 95% confidence intervals do not overlap, implying most likely significant differences between groups (despite the small sample sizes). Also, every observed value for FEP is above the intervals 3–4 and apparently always higher than HC. But, due to the small sample size per group, we performed beta regressions to test differences (refer to Table 2).

Table 2 shows beta regression coefficient estimates and their respective standard deviations as well as levels of significance for each of the measured factors (and factor levels): (i) modulation envelope (STH = sawtooth, SINE = sine), (ii) order (ASC = ascending, DESC = descending), (iii) duration (4s, 8s), and (iv) range (0.050–8kHz, 2–8kHz). Differences between groups (*p*<0.01) were found for both ascending and descending stimuli whose envelops were modulated by SINE. Sound discomfort level was set higher by FEP than by HC for all 16 stimuli. Also, significant differences between groups (*p*<0.05) were found for the factor levels STH, SINE, ASC, DESC, 2–8kHz, and 8 s. In every case, sound discomfort level (SDL) in FEP was found to be higher than HC (Fig. 3).

### Kruskal-Wallis ANOVA analysis

**Fig. 4:**
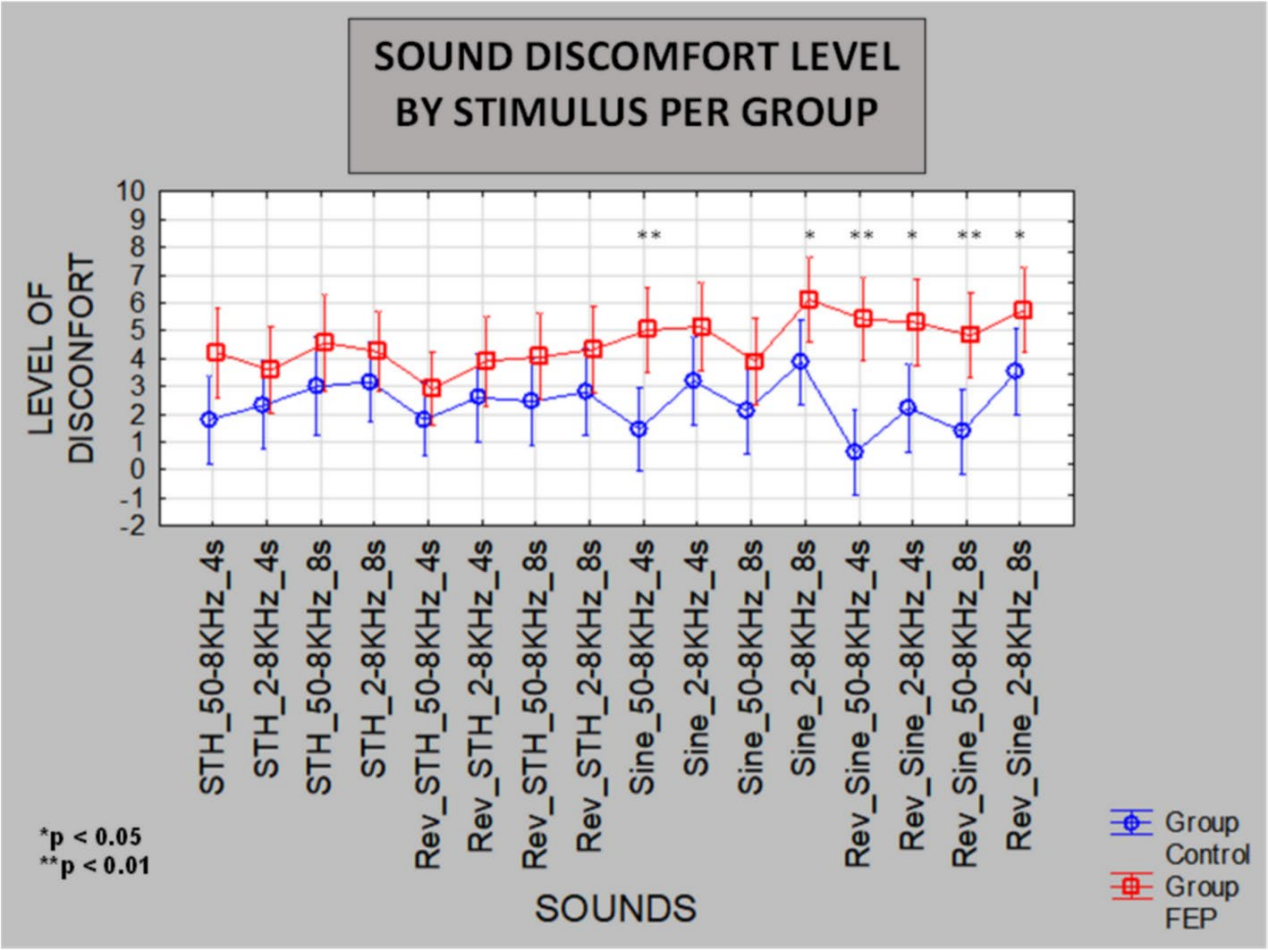
Observed mean SDL for FEP (red-square) and HC (blue-circle) for all 16 pure-tone frequency sweep sound stimuli. Levels of significance are given by Kruskal-Wallis ANOVA by Ranks for independent groups. In this case, significance was only attained for sounds modulated by SINE envelopes. Note that the mean SDL for FEP is always set above the observed mean levels for HC for every one of the 16 sounds

A non-parametric alternative statistical treatment less suitable to the observed data due to the small sample size was also carried out. Figure 4 shows a summary result for the 16 sound frequency sweeps. In this case, SDL was significantly higher in the FEP group for only six sounds, being all frequency sweeps modulated by SINE envelopes: two ASC and four DESC. That is, all DESC sound frequency sweeps modulated by SINE envelopes cause higher SDL (*p* < 0.05) in FEP, but only two ASC sounds (SINE, 50–8000Hz, 4s) and (SINE, 2–8kHz, 8s) caused significantly higher SDL in the same FEP group (*p* < 0.05), all in comparison to HC. Please note that the differences in SDL for combined ASC-SINE, and DESC-SINE, reached the level of significance *p* < 0.01 (refer to Table 2). While combined ASC-STH and DESC-STH did not yield observed significant differences between groups.

### Results from cognitive assessment

A summary of observed data from the Addenbroke Cognitive Examination is shown in Tables 3 and 4. On the one hand, in Table 3, we can observe that FEP and HC differed in the measurement estimates of *recognition (p<0.023)* and *visuospatial (p<0.034)* abilities that, in turn, strongly affected the *total score (p<0.009)*. The performance of FEP was worse in *recognition* than *visuospatial* ability, and both performances scored lower than estimates for HC. On the other hand, Table 4 shows the distribution of errors across the 16 items from this test. Errors are dispersed throughout the items, and more frequent in FEP. We counted as an observer’s error those answers that scored below 50% of the possible total of correct ones, within every 16 items of the test. The major errors of FEP were in *retrograde memory (7 people)*, *visuospatial ability (7 people)*, and *long-term recall and recognition (6 people).* The major errors were made by HC as well, that is, eight people scored below 50% correct in the item *anterograde memory*. Only three FEP patients scored lower than 50% correct in 7–10 items. The remaining 11 FEP patients scored over 50% correct answers in 11–16 of the 16 items.

**Table 3:** Summary of Addenbroke results

	Mean FEP	Mean HC	Maximum score	***P*** value
**Attention and orientation**	15.71	17.14	18.00	*p* = .126
**Memory**	10.86	11.57	14.00	*p* = .727
**Fluency**	9.86	11.79	14.00	*p* = .130
**Language**	24.14	25.50	26.00	*p* = .118
**Visuospatial**	12.00	14.43	16.00	*p* = .034*
**Recognition**	6.86	10.50	12.00	*p* = .023*
**Total score**	79.43	90.93	100.00	*p* = .009**

*Note.* We used Kruskal-Wallis* *p* < .05; **p < .01

**Table 4:** Summary errors in Addenbroke individual items

Addenbroke 16 items	Correct ≤ 50%	
	FEP^a^	HC^a^
**Recall memory**	3	1
**Retrograde memory**	7	0
**Anterograde memory**	2	8
**Fluency**	3	1
**Orientation**	0	0
**Registry**	0	0
**Attention & focus**	3	2
**Language comprehension (command)**	1	0
**Language comprehension (cathegory)**	1	1
**Language naming**	1	0
**Language reading**	1	0
**Language writing**	3	0
**Language repetition**	1	0
**Perceptual ability**	1	0
**Visuo-spatial perception*(p=0.0395)***	7^a^	3
**Long-term recall and recognition*(p=0.0005)***	6**	0
**Correct = 7–10 from 16 ITENS**	3	0
**Correct ≥ 11 from 16 ITENS**	11	14

*Note*. ^a^Number of volunteers that made errors ≥ 50%, **p < .01

### Spearman correlation analyses

There were no significant correlations between education, age, the Addenbroke Cognitive Examination (items or total), and SDLs for any one or all the 16 pure-tone frequency sweeps taken together.

On the other hand, responses to all 16 sounds were highly correlated among themselves, SDLs of both groups observed for the 16 sounds correlated with each one of the remaining 15 sounds (*p < 0.05)*, suggesting valid and consistent measurements of SDL for both FEP and HC.

## Discussion

Our study has measured the subjective quality of hearing perception in the tolerance/discomfort dimension in FEP and HC, and as expected, lower hearing tolerance, that is, higher SDL were observed for FEP in general (refer to Fig. 4). Some frequency sweeps did elicit higher SDL than others in FEP as did for HC. But six of the frequency sweeps modulated by sine envelopes strongly differentiated FEP from HC. On the other hand, apparently, no pure-tone frequency sweeps modulated by sawtooth envelopes did so.

Nevertheless, when considering results from the group comparisons based on the beta regression analysis, six of the eight factor levels differentiated FEP from HC: SINE, STH, ASC, DESC, 2–8 kHz, and 8s. In addition, combined ASC-SINE and DESC-SINE conditions were observed to elicit very high levels of discomfort (*p* < 0.01) in FEP in comparison to ASC-STH and DESC-STH conditions that did not show differences between groups.

Although we could consider discarding frequency sweeps modulated by STH envelopes, we must consider that half of these kind of stimulus has elicited mean SDL above 4 in the scale of 10 (refer to Fig. 4) and the fact that the factor level STH did differentiate FEP from HC as well. So, it should be further investigated with larger samples. Also, for that same reason, it may be too soon to discard the duration of 4s and the range 50–8000 Hz.

In sum, our results do support our hypothesis of existing auditory sensory hypersensitivity in FEP patients even though in treatment and medicated. This fact has been systematically overlooked and ignored in the literature (and the clinic). So, within the context of our comprehensive and exhaustive introduction, the present work brings a sensory and perceptual new dimension into schizophrenia research not previously endorsed, namely, hearing tolerance/discomfort due to auditory sensory hypersensitivity. This is a topic that does not appear in the current literature, neither fits into the previously reviewed topics. The closest evidence loosely linked to our present study is the issue of hearing sensitivity occasionally mentioned in the bulk of the literature of schizophrenia. However, we should recognize that hypersensitivity is indeed very difficult to assess and measure psychophysically and might be hidden within, and confounded with, tinnitus.

Further, most audiometric measurements, at least in Brazil, only cover the range up to 8 kHz, the upper limit of speech sounds. The work of Silva and Feitosa (2006), for instance, addresses the issue that “normal” audiometry does not cover the range beyond 8 kHz and shows the presence of significantly high sensitivity in the range between 8 and 16 kHz for young people (age < 50), as well as significant losses in sensitivity within this same range with increasing age (older people with age > 50).

Furthermore, a study just published in April 2022, by the group of Dean Salisbury (Coffman et al., 2022), using ASSR, points to the excessive basal excitability of the auditory cortex in FEP patients, a finding that is most consistent with our hypothesis of a conspicuous auditory sensory hypersensitivity in FEP and schizophrenia patients.

Despite its limitation due to small sample size and lack of patient detailed medical anamneses, or baseline audiometry, this study presents a test that might be very helpful in the assessment and diagnosis of people at risk for schizophrenia and FEP. We assume that this perceptual lack of tolerance, or discomfort, derives from the increased auditory sensitivity in early at-risk and prodromal states and precedes severe cognitive impairments, and thus, can be used to detect and prevent aggravation of psychosis related symptoms, and perhaps yet other related neuropsychiatric illnesses. Therefore, we propose that it be routinely included as part of psychiatric ambulatorial assessment. The issue of tinnitus, or the possibility of hidden auditory sensory hypersensitivity, mainly in mid and high frequency pure tones, should also be systematically addressed once it may be co-morbid in people developing FEP and schizophrenia.

We should also note that assessment with the Addenbroke Cognitive Examination did show a small difference in cognitive performances between FEP and HC (refer to Tables 3 and 4). A closer examination shows that those deficits are mostly concentrated in the errors of three patients from FEP, and they appear to have strongly contributed to lower the total score of the group. Finding a difference in some cognitive abilities between FEP and HC, as assessed by the Addenbroke Cognitive Examination, does not weaken our hypothesis of worsening sensory and perceptual capabilities prior to noticeable cognitive deficits in the progression of the prodromal and schizophrenic states. We firmly stand by it.

Also, we did not find significant correlations between the SAT and the Addenbroke Cognitive Examination, ACE-R, total score, neither with the isolated factor scores. Meaning that our findings are unrelated to the cognitive abilities assessed by ACE-R and, nevertheless, these were extremely helpful in successfully discriminating between FEP and HC. So, this is precisely the main contribution of the present study, the emphasis on the sensory-perceptual dimension, while placing the focus precisely on the conspicuous sensory hypersensitivity of the auditory system in FEP and early schizophrenia. It might wear out with chronic schizophrenia, as it does with increasing age. And there is evidence of auditory cortex loss in chronic schizophrenia (e.g., Hirano et al., 2020).

On the other hand, the only significant correlations we found were among the SDL responses from both groups to each of the sounds (highly correlated among themselves). This is evidence that favors the existence of internal consistency and validity of the SAT to estimate SDL (and to differentiate groups of people diagnosed with FEP (and most likely with schizophrenia too).

In resume, this shows that SDLs measured by the SAT are independent of cognitive factors (at least those evaluated by the Addenbroke Cognitive Examination) and very suitable as an additional sensory and perceptual test, being a tool for assessing FEP and possibly other neuropsychiatric disorders related to psychosis or schizophrenia.

Finally, we also strongly suggest that besides the usually considered factors to describe the disease schizophrenia, and FEP, that is, (i) positive symptoms, (ii) negative symptoms, and (iii) cognitive symptoms, we include (iv) sensory and perceptual symptoms as an additional, primary, and necessary classification that has the potential to play a key and central role in assessment to provide precocious diagnosis.

## Conclusion

The present work introduces a novel means of assessment to be included in the evaluation of people with psychotic symptoms seeking professional help. The “Sound Appreciation Test”, SAT, measures auditory hearing tolerance and discomfort to 16 pure tone sweep sound stimuli that we have shown to differentiate volunteers diagnosed with FEP from HC. This is a sensory test that did not correlate with cognitive symptoms.

We consider these results as evidence of an existing independent sensory-perceptual dimension not previously measured in the literature that, therefore, constitutes a genuine and original new factor to be noted and evaluated when psychophysically assessing neuropsychiatric patients. Besides, SAT is most likely very suitable to help diagnose and prevent aggravation of psychosis in prodromal states and FEP.

Based on both the present results and on our assumption that the worsening of perceptual symptoms in FEP, and schizophrenia, precedes the worsening of the cognitive symptoms, we suggested the inclusion of *sensory and perceptual symptoms* as an additional and necessary classification to assess those conditions.

## Data Availability

The datasets generated and/or analyzed during the current study are not publicly available due the rights of privacy regarding the volunteers for the experiments, but are available from the corresponding author on reasonable request.
